# Predictive factors of major amputation in patients with diabetic foot ulcers treated by peripheral blood mononuclear cells

**DOI:** 10.1007/s00592-025-02522-2

**Published:** 2025-05-27

**Authors:** Marco Meloni, Ermanno Bellizzi, Luigi Uccioli, Laura Giurato, Valeria Ruotolo, Martina Salvi, Federico Rolando Bonanni, Aikaterini Andreadi, Alfonso Bellia, Davide Lauro

**Affiliations:** 1 Department of Systems of Medicine, University of Tor Vergata, Viale Oxford 81, Rome, 00133 Italy; 2 Division of Endocrinology and Diabetology, Department of Medical Sciences, University Hospital Fondazione Policlinico Tor Vergata, Viale Oxford 81, Rome, 00133 Italy; 3 Department of Endocrinology and Diabetology, CTO Hospital, Rome, 00145 Italy

**Keywords:** Diabetes, Diabetic foot, Amputation, Peripheral arterial disease, Peripheral blood mononuclear cells

## Abstract

**Aim:**

Peripheral blood mononuclear cells (PB-MNCs) therapy is an adjuvant treatment for patients with ischaemic diabetic foot ulcers (DFUs) and no-option critical limb ischemia (NO-CLI). This study aimed to evaluate factors influencing the effectiveness of PB-MNC therapy.

**Method:**

This prospective, not controlled study included a cohort of patients with DFUs and NO-CLI treated by PB-MNCs. NO-CLI was defined as the revascularization failure with desert foot (absence of any artery below-the-ankle) or partial desert foot (absence of a wound-related artery with TcPO_2_ < 30 mmHg) at the final post-procedural angiogram. After one year of follow-up, the rate of major amputation was evaluated such as clinical, wound, and vascular features of amputees in comparison to not amputees. In addition, the factors influencing the risk of major amputation were analyzed.

**Results/discussion:**

Sixty-four patients were included. The mean age was 73.8 ± 5.8 years, 75% were male, and all of them had type 2 diabetes. At one year of follow-up, major amputation was documented in 12.5% of patients. Amputees had a higher rate of desert foot (vs. partial desert foot) (100% vs. 25%, *p* < 0.0001), higher post-procedural pain (5.7 ± 1.9 vs. 2.2 ± 1.3, *p* < 0.0001), lower TcPO_2_ after PB-MNCs therapy (30 ± 8 vs. 43 ± 8 mmHg, *p* = 0.0001), and more cases of heel ulcers (75% vs. 21.4%, *p* = 0.002). Independent predictors of major amputation resulted the presence of desert foot, persistence of post-procedural pain, heel involvement with multiple ulcers, and inability to stand or walk without assistance.

**Conclusion:**

PB-MNCs therapy resulted less effective in patients with complete desert foot, persistence of paint after therapy, heel involvement in persons with multiple ulcers, and impaired walking.

## Introduction

The last decades have documented an evolution of patients with diabetic foot ulcers (DFUs) by the increase of ischaemic subjects in comparison to the neuropathic subjects [1]. Recent data have showed that ischaemic DFUs involve nearly 70% of diabetic patients who referred for a new foot ulcer to a dedicated diabetic foot service [2], being a great burden for clinicians and health care systems.

Even though many goals have been achieved through significant improvements in lower limb revascularization in patients with ischaemic DFUs and critical limb threatening ischaemia (CLTI) [3], there is still a significant rate of patients in which vascular procedures are unsuccessful or not feasible. Nowadays, it is counted the 15–25% of patients have a partial or total failure of lower limb revascularization due to the inability to overcome stenosis or obstruction of leg/foot arteries [4]. Unsuccessful revascularization failure increases the risk of major amputation up to 30% [4] and this group of patients are termed as no-option critical limb ischaemia (NO-CLI) subjects.

NO-CLI patients are characterized by the presence of several co-morbidities such as end-stage-renal-disease (ESRD) and ischamic heart disease (IHD), a multi-level vascular disease and a frequent involvement of below-the-ankle (BTA) arteries (> 70% of cases) [4].

In the last years, the use of autologous cell therapy (ACT), specifically peripheral blood mononuclear cells (PB-MNCs) therapy, in NO-CLI patients has achieved promising results by reducing the rate of major amputation and mortality [5, 6].

PB-MNCs includes various cell types such as stem cells and immune cells, which have regenerative properties. This type of autologous cells can potentially enhance local angiogenesis, the formation of new blood vessels, thereby improving tissue perfusion and oxygenation in the wound angiosome area [7, 8]. Moreover, PB-MNCs seem to play a key role in tissue regeneration promoting the polarization from inflammatory macrophage M1 to the M2 regenerative phenotype [7, 8].

PB-MNC therapy has been widely used in different settings, in patients with different characteristics and often without not so close homogeneous criteria among previous clinical studies.

Nonetheless, there are very few data to well understand which patients are clearly suitable for having the best effectiveness through PB-MNCs therapy and which factors could influence the clinical response.

Accordingly, the current study aimed to evaluate the predictive factors influencing the response to PB-MNCs therapy with the aim to identify which patients are more eligible for receiving this procedure and achieve better outcomes.

## Materials and methods

The current study is a prospective non-controlled study including a population of diabetic patients affected by neuro-ischaemic DFUs and NO-CLI managed between March 2019 to December 2022 in the Department of Endocrinology, Diabetology and Metabolic disease, a tertiary level diabetic foot service, at the University Polyclinic of “Tor Vergata” serving Rome, Italy.

Included patients were those with desert foot (absence of any vessels below-the-ankle such as pedal artery and plantar arteries) and/or those with failed direct revascularization of “wound related artery” with persistent foot ischemia in the wound angiosome area defined by TcPO2 levels < 30 mmHg [9]. The evaluation of foot artery disease, so the presence of desert foot or partial desert foot (failed direct revascularization of “wound related artery”) has been defined by angiographic criteria after the evaluation of post-procedural angiograms Table 1.

**Table 1:** Inclusion and exclusion criteria for the study enrolment

Inclusion criteria	Exclusion criteria
Ischaemic/neuro-ischaemic DFU stage 1 C-D, 2 C-D, 3 C-D according to Texas University Classification	Indication to primary amputation due to absence of anatomical suitability for surgical foot salvage
Unsuccessful revascularization below-the-ankle: desert foot and/or absence to direct flow to the wound angiosome area with TcPO2 < 30 mmHg	Absence of any vessels below-the-knee
Patency of at least on vessel below-the-knee (anterior tibial artery, posterior tibial artery, peroneal artery)	Reduced life expectancy (less than 6 month)
	Immunosuppressive therapy
	Active neoplastic disease
	Severe cognitive impairment
	Lost to follow-up

DFU: diabetic foot ulcer

In addition, only patient with at least a patent artery below-the-knee (anterior tibial artery, peroneal artery and posterior tibial artery) were considered for the administration of ACT. Included patients were also those with ischaemic/neuro- ischaemic DFUs belonging to stage 1 C, 2 C, 3 C and 1D, 2D, 3D according to Texas University Classification [10]. Inclusion and exclusion criteria were reported in Table 1.

All patients were managed by a multi-disciplinary foot team through a pre-set limb salvage protocol based on the International Working Group on the Diabetic Foot (IWGDF) guidelines including antibiotic therapy (and surgery if required) in the case of infection, offloading of affected foot, local wound care, and management of concomitant co-morbidities [11, 12]. 

At the baseline demographic, clinical and wound characteristics were recorded.

### Clinical features

Hypertension was considered if the patient was currently receiving anti-hypertensive therapy or in the case of blood pressure > 140/90 mmHg. Hypercholesterolemia was defined in the case of statin therapy assumption or elevated low-density lipoprotein (LDL) level at the assessment (> 55 mg/dl) requiring statin therapy. Patients with a history of an acute coronary syndrome, prior revascularization for ischaemic heart disease (IHD), typical angina or suggestive electrocardiographic findings (such as ST elevation, ST depression, pathological Q waves, T wave inversion) or new left bundle branch block were considered to have IHD. A diagnosis of heart failure was made on the basis of typical symptoms and echocardiographic findings, including LVEF < 40% (or normal/mildly reduced LVEF) with elevated biomarkers BNP > 35 pg/ml and/or NT-proBNP > 125 pg/ml not explainable by other causes a non-dilated left ventricle associated with significant structural heart disease (e.g.: left ventricular hypertrophy or left atrial enlargement), and/or diastolic dysfunction. Smoker status was recorded as a baseline characteristic if the patient reported smoking at the time of assessment. Patients with history of cerebrovascular ischemia, previous carotid artery revascularization or new diagnosis of atherosclerotic plaque occluding more than 70% of carotid arteries were considered as patients with cerebrovascular disease. Additionally, the rate of patients with end-stage renal disease (ESRD) requiring dialysis was reported as well as the distribution of patients in the different classes of chronic kidney disease.

### Wound assessment

Wounds features were recorded at the first assessment according to IWGDF definitions [11]. Diagnosis of infection has been done according to clinical signs (redness, warmth, swelling, induration, tenderness, pain, purulent secretion). Osteomyelitis was considered in the case of deep ulcer involving the bone, confirmed by radiological evaluation (x-ray or magnetic resonance) and positive microbiological analysis [13]. 

### Vascular assessment and definition and cell therapy procedure

Technical failure of revascularization procedures was defined as the inability to achieve successful recanalization of stenosed or occluded vessel specifically not allowing an adequate blood flow to the foot in the wound angiosome area.

In the case of unsuccessful peripheral revascularization, included patients received the adjuvant cell-therapy using a concentrated solution of autologous PB-MNCs in the affected limb. PB-MNCs used in the recruited population was produced by point of care HemaTrate ^®^ Blood Filtration System (Cook Regentec Indianapolis, Indiana–USA). The cell product obtained by gravity filtration has been previously extensively characterized in terms of composition, recovery, and cytofluorimetric cell population analysis [14].

One hundred and twenty ml of acid-citrate-dextrose (ACD)-anticoagulated peripheral blood was loaded in the upper blood bag and gravity filtration was allowed. The captured MNCs were harvested by sterile saline back- flush and immediately implanted. All the procedures were performed in the operatory/surgery room by an anaesthesiologic support if retained or the use of a peripheral block.

PB-MNCs were administered through a “below-the-ankle approach” in the affected foot along the wound related artery according to the angiosome theory [15]. The treatment was repeated for three times 21–42 days apart. Within 2–4 weeks after the last PB-MNC treatment, all patients received the surgical treatment (toe amputation, ray amputation, sequestrectomy, bone resection, trans-metatarsal amputation, etc.) based on each specific clinical framework, otherwise the local treatment was performed by secondary intention according to the characteristics of the target ulcer.

The foot blood perfusion, specifically in the wound angiosome area, was evaluated through TcPO2 before starting PB-MNCs therapy and 2–4 weeks after the last procedure. At the same time, the foot pain before starting PB-MNCs therapy and 2–4 weeks after the last procedure was evaluated through the numerical rating scale (NRS).

The study was performed according to the Declaration of Helsinki and was approved by the Ethics Committee (protocol number MC-01-2016; 151/16).

### Outcome measures

After 1-year of follow-up the rate of major amputation was reported. Accordingly, patients were divided in two groups, amputees and not amputees. Baseline characteristics were compared between groups. In addition, predictive factors of major amputation were analysed.

### Statistical analysis

Statistical analysis was conducted using SAS software (JMP12; SAS Institute, Cary, NC). Data were presented as means ± standard deviation (SD). Univariate logistic regression analysis was employed to assess all potential predictor variables, with odds ratios (ORs) and 95% confidence intervals (CIs) provided to evaluate their association with the outcome of interest (major amputation). Subsequently, all potential predictors of outcome were included simultaneously in a multivariate logistic regression model, which identified the variables that most effectively predicted the outcomes. A p-value of less than 0.05 was considered statistically significant for all analyses.

## Results

Overall, 64 patients were included in the study. The majority of them were aged (> 70 years), with a great prevalence of males. All participants were diagnosed with type 2 diabetes with a long diabetes duration exceeding 20 years. Several co-morbidities were found in the whole population, including specifically a history of IHD, hypertension, and dyslipidemia. For that concerning wound characteristics, the majority were infected (approximately 81%), 93%, had a gangrene, 30% had a heel location. Regarding the vascular characteristics, 34.4% of patients reported a condition of desert foot, while 65.6% had a wound angiosome ischaemia after receiving indirect revascularization. Table 2.

**Table 2:** Comparison between amputees and not amputees

Variable	Whole population *n* (%) 64 (100%)	Amputees *n* (%) 8 (12.5%)	Not amputees *n* (%) 56 (87.5%)	*p*-value
Age (Years)	73,8 ± 5,8	76.5 ± 4.7	73.4 ± 6	0.1
Sex (Male) n (%)	48 (75)	6 (75)	42 (75)	1
Diabetes (Type 2)	64 (100)	8 (100)	56 (100)	1
Diabetes Duration (Years)	21.6 ± 6	26 ± 5	21 ± 6.7	0.08
Hba1c mmol/mol (%)	43 ± 23 (6.1 ± 4.3)	45 ± 21 (6.3 ± 4.1)	43 ± 23 (6.1 ± 4.3)	0.2
Anemia	22 (34)	6 (75)	16 (28.6)	0.01
Hypertension	64 (100)	8 (100)	56 (100)	1
IHD	56 (87.5)	6 (75)	50 (89.3)	0.2
Cerebrovascular Disease	42 (65.6)	8 (100)	34 (60.7)	0.006
Active Smoke	10 (15.6)	0 (0)	10 (17.8)	0.08
Heart Failure	8 (12.5)	2 (25)	6 (10.7)	0.03
ESRD	0 (0)	0 (0)	0 (0)	1
CKD class 3	38 (59.4)	5 (62.5%)	33 (58.9)	0.4
CKD class 4	17 (26.5)	2 (25%)	15 (26.7)	0.2
CKD class 5	1 (1.5)	0	1 (1.8)	0.5
Diabetic Retinopathy	54 (84.4)	8 (100)	46 (82.2)	0.08
Inability to stand or walk without help	12 (18.7%)	4 (50%)	8 (14.2%)	< 0.0001
Ulcer Size (> 5 Cm^2^)	52 (81.2)	8 (100)	44 (78.6)	0.05
Infection	52 (81.2)	8 (100)	44 (78.6)	0.05
Osteomyelitis	32 (50)	4 (50)	28 (50)	1
Gangrene	60 (93.7)	8 (100)	52 (92.8)	0.2
Heel involvement in association to multiple ulcers	18 (28.1)	6 (75)	12 (21.4)	0.002
Desert Foot	22 (34.4)	8 (100)	14 (25)	< 0.001
Pedal Disease	48 (75)	8 (100)	40 (71.4)	0.02
Plantar Arteries Disease	50 (78.1)	8 (100)	42 (75)	0.03
Baseline TcPO2 (mmHg)	17.6 ± 6	10 ± 4	18 ± 5	0.0003
TcPO2 After Treatment (mmhg)	41 ± 8	30 ± 8	43 ± 8	0.0001
Baseline Pain (NRS)	7.1 ± 1.8	7.2 ± 2.3	7.1 ± 1.8	0.8
Pain (NRS) After Treatment	2.6 ± 1.3	5.7 ± 1.9	2.2 ± 1.3	< 0.0001

IHD: Ischaemic heart disease; ESRD: end-stage-renal-disease; CKD: chronic kidney disease; TcPO2: transcutaneous oxygen pressure; NRS: numerical rating scale

At the one-year follow-up, 71.9% of patients healed and survived, 3.1% healed and died, 9.4% did not heal and died, 12.5% unhealed and underwent major amputation, and 3.1% unhealed without requiring major amputation. Comparing the group of patients who received major amputation and those who achieved limb salvage, amputees showed a higher rate of heel location, higher rate of desert foot, higher post-procedural pain, and lower TcPO2 values after PB-MNCs therapy. Table 2.

At the multivariate analysis the following factors resulted independently related to a poor response to PB-MNCs therapy and increased risk of major amputation: the presence of desert foot (in comparison to the presence of residual wound angiosome ischaemia after indirect revascularization), the persistence of post-procedural pain requiring analgesic therapy, the heel involvement in association to multiple ulcers, and the inability to stand or walk without help. Table 3.

**Table 3:** Multivariate analysis on outcomes of interest of independent factors found at the univariate analysis

Variables	Major amputation		
	OR	95% CI	*p* value
Desert foot (vs. indirect revascularization)	3.7	1.2–7.2	0.0002
Persistence of post procedural pain	2.8	1.1–4.8	0.0006
Multiple ulcers with heel location	2.8	1.7–4.1	0.008
Inability to stand or walk without help	3.9	1.5–8.6	0.0002
Heart failure	1.4	0.8–1.9	0.6

## Discussion

This study confirms the potential effectiveness of PB-MNCs therapy in NO-CLI patients who have a failure of mechanical revascularization procedure. The current data reported approximately 72% of healing and 12.5% of major amputation at 1 year of follow-up.

Even though the rate of major amputation seems to be higher when compared to patients who receive successful revascularization, the same appears significantly lower when compared to NO-CLI treated by conventional therapy (12.5% vs. 30%) [4]. 

The rate of major amputation in our population is more or less similar to that found in other studies evaluating the effectiveness of PB-MNCs therapy. Monami and Panunzi found respectively 20% and 18% of major amputation in a cohort of ischaemic diabetic foot subjects not treatable by conventional revascularization procedure [16, 17]. Scatena et al. found 10.5% of major amputation at 2 years of follow-up in patiens managed by PB-MNCs therapy in comparison to 39.5% of major amputation in the control group managed by conventional therapy [18].

The main aim of authors was to better understand the characteristics of amputees and which factors could influence the effectiveness of ACT. The group of patients receiving major amputation did not show different clinical characteristics when compared to not amputees, except for the presence of cerebrovascular disease and heart failure which were more present in patients who experienced major amputation. Otherwise, amputees seem to present a more aggressive vascular disease: they had in all cases a concomitant arterial disease of pedal and plantar arteries (desert foot at the final angiogram), lower TcPO2 values at the baseline (10 vs. 18 mmHg) and after cell therapy (30 vs. 43 mmHg), and higher foot pain after cell therapy (5.7 vs. 2.2 evaluated through NRS).

The concomitant absence of blood supply from pedal artery and plantar arteries (medial and lateral), so termed “desert foot”, is the most severe condition of CLTI and a documented independent risk factor for amputation [19, 20]. The absence of any vessel BTA leads to a risk of major amputation up to 40% [20] and BTA revascularization is mandatory to achieve limb salvage and wound healing. In addition, the presence of “desert foot” resulted an independent risk factor of less effectiveness of cell therapy when compared to the condition of partial “desert foot” in which cell therapy was used as adjuvant therapy for patients showing a residual wound angiosome ischaemia after receiving indirect revascularization. These data reflect what already documented in recent previous studies in which cell therapy was greatly effective in patients receiving indirect revascularization where any subject received major amputation [21], while 16.4% of major amputation was documented in those patients with complete “desert foot” treated by cell therapy [15]. These elements highlight that the presence of at least one good vessel BTA, even if not perfusing the wound angiosome area, could be a rescue vessel reducing the severity of ischaemia and allowing higher benefits from PB-MNCs therapy which can help to reconstruct small arteries and the foot vascular network [7, 17]. The other two vascular variables which highlight the potential less effectiveness of cell therapy in patients with “desert foot” receiving amputation are related to the foot oxygenation defined by TcPO2 parameters and the local pain. In amputees, TcPO2 values are significantly lower in patients with “desert foot” and even though cell therapy allowed an evident increase of blood perfusion (from 10 to 30 mmHg), the vascular improvement could be in some cases not enough for saving the foot, especially in the case of extended gangrene involving soft tissues and bone. In addition, the persistence of foot pain requiring medical therapy reflects only a partial improvement of foot ischaemia. The persistence of a severe or moderate ischaemia not allowing a demolitive/reconstructive surgical procedure in some specific cases, and the persistence of local pain resistant to the medical therapy and impairing the quality of life, can require a major amputation as usual happens in our clinical experience.

The presence of multiple ulcers/gangrene with the involvement of the heel and the inability to stand or walk without help resulted the other factor influencing the risk of major amputation. The presence of ulcers located in different parts/angiosomes of the foot usually require the presence of sufficient blood perfusion in different anatomical area of the foot and the paracrine vascular activity of autologous cell therapy could be not completely sufficient to ensure the same vascular support for each angiosome, especially in the case of heel ulcer. Heel ulcers are the most difficult ulcers to be treated with less chance of healing and longer healing time when compared to ulcer located in fore and midfoot [22–24]. This element is usually related to the anatomical characteristics of the heel defined by a poor vascular perfusion also in physiological conditions (due to the absence of muscle tissue) and the low chance to be treated by 1st intention (absence of adequate elastic tissue for covering post-surgical wounds).

The inability to stand or walk without assistance is a well-known independent factor for non-healing [25]. This condition appears often associated to a patient’s frailty, resulting in the absence or poor amount of muscle tissue. The lack of adequate muscle tissue reduces the chances of PB-MNCs being effective in such cases, requiring vascular remodeling a sufficient muscle scaffold [7].

As we found in our analysis, Pan et al. found that non-responders to cell therapy showed lower TcPO2 values in comparison to responders [26]. This data can be easily related to the poor presence of oxygen in the wound angiosome area that impair the ability of healing process. The same research group identify also some other factors influencing the outcomes, specifically age (> 50 yrs), blood fibrinogen (> 4 g/L), arterial occlusion level (above-the-knee), and the total transplanted CD34^+^ cell count [26]. Similarly, Panunzi et al. reported that patients with high level of TcPO_2_ (≥ 40 mmHg) showed better outcomes in terms of healing and limb salvage [17]. Patients with higher TcPO2 level presented also a higher frequency of extracellular vesicles at the enrollment, which are known markers of angiogenesis [17].

A real interesting paper of Dubsky et co-researchers analysed some factors influencing a successful response to ACT. The main independent predictors of impaired response to ACT were found on some thrombotic disorders, specifically the presence of heterozygote Leiden mutation and homozygote methylenetetrahydrofolate reductase (MTHFR 677) mutation [27].

Definitely, our data seem to highlight as the severity of foot ischaemia at baseline (desert foot, lower TcPO2 values), the characteristics of tissue loss (multiple ulcers with heel location) and patients’ frailty could influence the response to PB-MNCs therapy. These items reflect some data documented in previous studies in which the severity of vascular disease at the assessment influenced the outcomes of ACT. Nonetheless, the study documented the effectiveness of PB-MNCs in patients with NO-CLI and its general effectiveness in terms of limb salvage as widely reported by the most important research in the field [28, 29].

To the best of our knowledge the current study is the first one to analyse different clinical and vascular factors influencing the response to PB-MNCs therapy. The population included is homogeneous such as the protocol of treatment of each patient, allowing a good overview of responders and non-responders (or poor responders) in the daily clinical practice.

The use of ACT is now considered a useful adjuvant therapy in some extreme vascular conditions, although it is only a specific part of a global treatment, requiring patients with DFUs a multidisciplinary team approach for allowing good outcomes in terms of healing, limb preservation and survival [30].

The study has some limitations. It is a monocentric study, and the sample is not so large even if adequate when compared to similar studies. Some potential influencing factors such as haematologic and thrombotic disorders are not analysed and combined with clinical factors. At the same time, angiogenic markers at the baseline and the response to ACT have not been evaluated, therefore authors cannot differentiate clinical response from biological responses.

## Conclusions

The study highlights that different factors could influence clinical outcomes of patients with NO-CLI and DFUs treated by PB-MNCs, including vascular factors, wound characteristics, and patient general status. These data can help clinicians to better identify which patients are easily suitable for ACT and which could have poorer results, driving the potential surgical curative approach.

Further studies will be useful to identify the missing variable such as biological and hematological factors that could impact on paracrine activity and vascular remodeling by using cell therapy.

## References

[1] Meloni M, Piaggesi A, Uccioli L (2024) From a spark to a flame: the evolution of diabetic foot disease in the last two decades. Int J Low Extrem Wounds. 10.1177/15347346241238480 38470358 10.1177/15347346241238480

[2] Meloni M, Izzo V, Giurato L, Lázaro-Martínez JL, Uccioli L (2020) Prevalence clinical aspects and outcomes in a large cohort of persons with diabetic foot disease: comparison between neuropathic and ischemic ulcers. J Clin Med 9(6):1780 32521700 10.3390/jcm9061780 PMC7356179

[3] Lazzarini PA, Raspovic KM, Meloni M, van Netten JJ (2024) A new declaration for Feet’s sake: halving the global diabetic foot disease burden from 2–1% with next generation care. Diabetes Metab Res Rev 40(3):e3747 37997627 10.1002/dmrr.3747

[4] Meloni M, Izzo V, Da Ros V, Morosetti D, Stefanini M, Brocco E, Giurato L, Gandini R, Uccioli L (2020) Characteristics and outcome for persons with diabetic foot ulcer and No-Option critical limb ischemia. J Clin Med 9:3745 33233329 10.3390/jcm9113745 PMC7700155

[5] Dubský M, Jirkovská A, Bem R, Nemcová A, Fejfarová V, Jude EB (2017) Cell therapy of critical limb ischemia in diabetic patients—state of art. Diabetes Res Clin Pract 126:263–271 28288436 10.1016/j.diabres.2017.02.028

[6] Dubský M, Husáková J, Sojáková D, Fejfarová V, Jude EB (2023) Cell therapy of severe ischemia in people with diabetic foot Ulcers-Do we have enough evidence? Mol Diagn Ther 27(6):673–683 37740111 10.1007/s40291-023-00667-w PMC10590286

[7] Rehak L, Giurato L, Meloni M et al (2022) The immune-centric revolution in the diabetic foot: monocytes and lymphocytes role in wound healing and tissue regeneration-a narrative review. J Clin Med 11(3):889 35160339 10.3390/jcm11030889 PMC8836882

[8] Rehak L, Giurato L, Monami M, Meloni M, Scatena A, Panunzi A, Manti GM, Caravaggi CMF, Uccioli L (2024) The immune-centric revolution translated into clinical application: Peripheral Blood Mononuclear Cell (PBMNC) therapy in diabetic patients with No-Option Critical Limb-Threatening Ischemia (NO-CLTI)-rationale and meta-analysis of observational studies. J Clin Med. 13(23):7230 10.3390/jcm13237230 PMC11642624 39685690

[9] Norgren L, Hiatt WR, Dormandy JA et al (2007) TASC II Work- Ing group. Inter-society consensus for the management of peripheral arterial disease (TASC II). J Vasc Surg 45:S5–S67 17223489 10.1016/j.jvs.2006.12.037

[10] Armstrong DG (1996) The university of Texas diabetic foot classification system. Ostomy Wound Manag 42:60–61 8974409

[11] Schaper NC, van Netten JJ, Apelqvist J, Bus SA, Hinchliffe RJ, Lipsky BA, IWGDF Editorial Board (2020) Practical guidelines on the prevention and management of diabetic foot disease (IWGDF 2019 update). Diabetes Metab Res Rev 36(Suppl 1):e3266 32176447 10.1002/dmrr.3266

[12] Meloni M, Andreadi A, Bellizzi E, Giurato L, Ruotolo V, Romano M, Bellia A, Uccioli L, Lauro D (2023) A multidisciplinary team reduces in-hospital clinical complications and mortality in patients with diabetic foot ulcers. Diabetes Metab Res Rev 39(7):e3690 37422897 10.1002/dmrr.3690

[13] Lipsky BA, Senneville É, Abbas ZG, Aragón-Sánchez J, Diggle M, Embil JM, Kono S, Lavery LA, Malone M, van Asten SA, Urbančič-Rovan V, Peters EJG, International Working Group on the Diabetic Foot (IWGDF) (2020) Guidelines on the diagnosis and treatment of foot infection in persons with diabetes (IWGDF 2019 update). Diabetes Metab Res Rev 36(Suppl 1):e3280 32176444 10.1002/dmrr.3280

[14] Spaltro G, Straino S, Gambini E, Bassetti B, Persico L, Zoli S, Zanobini M, Capogrossi MC, Spirito R, Quarti C et al (2015) Characterization of the pall celeris system as a point-of-care device for therapeutic angiogenesis. Cytotherapy 17:1302–1313 26038175 10.1016/j.jcyt.2015.04.006

[15] Meloni M, Giurato L, Andreadi A, Bellizzi E, Bellia A, Lauro D, Uccioli L (2023) Peripheral blood mononuclear cells: a new frontier in the management of patients with diabetes and No-Option critical limb ischaemia. J Clin Med 12(19):6123 37834766 10.3390/jcm12196123 PMC10573900

[16] Ragghianti B, Berardi BM, Mannucci E et al (2023) Autologous peripheral blood mononuclear cells in patients with small artery disease and diabetic foot ulcers: efficacy, safety, and economic evaluation. J Clin Med 12(12):4148 37373842 10.3390/jcm12124148 PMC10298945

[17] Panunzi A, Madotto F, Sangalli E et al (2022) Results of a prospective observational study of autologous peripheral blood mononuclear cell therapy for no-option critical limb-threatening ischemia and severe diabetic foot ulcers. Cardiovasc Diabetol 21(1):196 36171587 10.1186/s12933-022-01629-y PMC9516816

[18] Scatena A, Petruzzi P, Maioli F, Lucaroni F, Ambrosone C, Ventoruzzo G, Liistro F, Tacconi D, Di Filippi M, Attempati N, Palombi L, Ercolini L, Bolognese L (2021) Autologous peripheral blood mononuclear cells for limb salvage in diabetic foot patients with No-Option critical limb ischemia. J Clin Med 10(10):2213 34065278 10.3390/jcm10102213 PMC8161401

[19] Meloni M, Izzo V, Giurato L, Gandini R, Uccioli L (2019) Below-the-ankle arterial disease severely impairs the outcomes of diabetic patients with ischemic foot ulcers. Diabetes Res Clin Pract 152:9–15 31078668 10.1016/j.diabres.2019.04.031

[20] Meloni M, Morosetti D, Giurato L, Stefanini M, Loreni G, Doddi M, Panunzi A, Bellia A, Gandini R, Brocco E, Lazaro-Martinez JL, Lauro D, Uccioli L (2021) Foot revascularization avoids major amputation in persons with diabetes and ischaemic foot ulcers. J Clin Med 10(17):3977 34501432 10.3390/jcm10173977 PMC8432560

[21] Marco M, Luigi U, Valeria R, Ermanno B, Carlo M, Maria R, Aikaterini A, Laura G, Alfonso B, Davide L (2024) Effectiveness of autologous mononuclear cells as adjuvant therapy in patients with ischaemic diabetic foot ulcers receiving indirect lower limb revascularization. Acta Diabetol. Sep 17. Erratum in: Acta Diabetol. 2024 Nov 7 10.1007/s00592-024-02375-1 39287795

[22] Örneholm H, Apelqvist J, Larsson J, Eneroth M (2017) Heel ulcers do heal in patients with diabetes. Int Wound J 14(4):629–635 27487819 10.1111/iwj.12654 PMC7950186

[23] Meloni M, Izzo V, Giurato L, Brocco E, Gandini R, Uccioli L (2020) Limb salvage in diabetic patients with ischemic heel ulcers. Int J Low Extrem Wounds 19(3):275–281 31744357 10.1177/1534734619884438

[24] Pickwell KM, Siersma VD, Kars M, Holstein PE, Schaper NC (2013) Eurodiale consortium. Diabetic foot disease: impact of ulcer location on ulcer healing. Diabetes Metab Res Rev 29(5):377–383 23390115 10.1002/dmrr.2400

[25] Prompers L, Schaper N, Apelqvist J, Edmonds M, Jude E, Mauricio D, Uccioli L, Urbancic V, Bakker K, Holstein P, Jirkovska A, Piaggesi A, Ragnarson-Tennvall G, Reike H, Spraul M, Van Acker K, Van Baal J, Van Merode F, Ferreira I, Huijberts M (2008) Prediction of outcome in individuals with diabetic foot ulcers: focus on the differences between individuals with and without peripheral arterial disease. EURODIALE Study Diabetologia 51(5):747–755 18297261 10.1007/s00125-008-0940-0 PMC2292424

[26] Pan T, Liu H, Fang Y, Wei Z, Gu S, Fang G, Liu Y, Luo Y, Guo D, Xu X, Chen B, Jiang J, Yang J, Shi Z, Zhu T, Shi Y, Liu P, Dong Z, Fu W (2019) Predictors of responders to mononuclear stem cell-based therapeutic angiogenesis for no-option critical limb ischemia. Stem Cell Res Ther 10(1):15 30635050 10.1186/s13287-018-1117-5 PMC6329149

[27] Dubský M, Fejfarová V, Bem R, Jirkovská A, Nemcová A, Sutoris K, Husáková J, Skibová J, Jude EB (2021) Main factors predicting nonresponders to autologous cell therapy for critical limb ischemia in patients with diabetic foot. Angiology 72(9):861–866 33783233 10.1177/00033197211005614

[28] Scatena A, Apicella M, Mantuano M, Ragghianti B, Silverii A, Miranda C, Monge L, Uccioli L, Scevola G, Stabile E, Gargiulo M, Vermigli C, Monami M (2024) Panel of the Italian guidelines for the treatment of diabetic foot syndrome and on behalf of SID and AMD. Autologous cell therapy for ischemic diabetic foot: a meta-analysis of randomized controlled trials for the development of the Italian guidelines for the treatment of diabetic foot syndrome. Acta Diabetol. 10.1007/s00592-024-02393-z. Epub ahead of print. Erratum in: Acta Diabetol. 2025 Jan 24. 10.1007/s00592-025-02455-w39724338

[29] Pu H, Huang Q, Zhang X, Wu Z, Qiu P, Jiang Y, Wang R, Zhao Z, Xu Z, Qin J, Lu X, Li W (2022) A meta-analysis of randomized controlled trials on therapeutic efficacy and safety of autologous cell therapy for atherosclerosis obliterans. J Vasc Surg 75(4):1440–1449 (**e5**) 34788653 10.1016/j.jvs.2021.10.051

[30] Meloni M, Giurato L, Monge L, Miranda C, Scatena A, Ragghianti B, Silverii GA, Vermigli C, De Cassai A, Volpe A, Tramonta R, Medea G, Bordieri C, Falcone M, Stefanon L, Bernetti A, Cappella C, Gargiulo M, Lorenzoni V, Scevola G, Stabile E, Da Ros R, Murdolo G, Bianchini E, Gaggia F, Gauna C, Romeo F, Apicella M, Mantuano M, Monami M, Uccioli L (2024) Panel of the Italian guidelines for the treatment of diabetic foot syndrome and on behalf of SID and AMD. Effect of a multidisciplinary team approach in patients with diabetic foot ulcers on major adverse limb events (MALEs): systematic review and meta-analysis for the development of the Italian guidelines for the treatment of diabetic foot syndrome. Acta Diabetol 61(5):543–553 38461443 10.1007/s00592-024-02246-9

